# Insects: Functional Morphology, Biomechanics and Biomimetics

**DOI:** 10.3390/insects12121108

**Published:** 2021-12-12

**Authors:** Hamed Rajabi, Jianing Wu, Stanislav Gorb

**Affiliations:** 1 Division of Mechanical Engineering and Design, School of Engineering, London South Bank University, London SE1 0AA, UK; 2 School of Aeronautics and Astronautics, Sun Yat-sen University, Guangzhou 510006, China; 3 Functional Morphology and Biomechanics, Zoological Institute, Kiel University, D-24118 Kiel, Germany

## 1. Introduction to the Special Issue

Insects are the most diverse animal taxon, both in terms of the number of species and the number of individuals. There are roughly one million described insect species, and their real number is estimated to be five to ten times this figure [1]. The total number of individual insects, on the other hand, is estimated to be as high as one million trillion [2]. This is why insects are regarded as the most successful groups of animals on Earth.

It is not the first time, nor will it be the last, that insects have become the core theme for a collection of experimental studies. However, what makes the current collection unique is the focus on understanding the complexities of insect structures, functions and potential applications that they offer. Although the functional morphology of insects remains a basic science, insect structures offer a variety of existing and potential applications in biomedical, structural, mechanical and aerospace engineering. In this Special Issue “Insects: Functional Morphology, Biomechanics and Biomimetics”, we aimed to include studies that cover fundamental research and discuss practical applications, as much as possible. 

In this Special Issue, our readers will read about insect structures, including wings, legs, feeding apparatus, sensory organs and ovipositors. The reader will find answers to questions such as: How do insects fly? What are the design strategies that enable insect wings to reach automatic shape control? How does the specific segmented design determine the oscillatory response of insect antennae? What are the adaptations of insect mouthparts to their feeding habits? How does a thin film of adhesive secretion contribute to attachment of insect eggs? What are the physiological and environmental factors that determine the jump performance of locusts? How can these inspire engineering innovations, such as wings for micro air vehicles, enhanced sensory systems or multifunctional microfluidic transporters?

A number of well-known scholars in the field kindly accepted our invitation and contributed to this collection. We would like to thank the authors for their invaluable contributions to this Special Issue. We appreciate their dedication, support and commitment. We would also like to thank the support of *Insects*, MDPI and its staff, who made this Special Issue possible. We very much thank the reviewers who assessed the submitted manuscripts and played a key role in improving the quality of this Special Issue. We hope that you enjoy reading this Special Issue.

## 2. Dedication

This Special Issue is dedicated to Professor Leonid I. Fransevich, a corresponding member of the Ukrainian National Academy of Sciences and Professor Emeritus at Schmalhausen Institute of Zoology, Kiev, Ukraine, for his work in insect functional morphology, physiology and biomechanics, and on the occasion of his 85th birthday.

Leonid Frantsevich was born in 1935 in Kiev, Ukraine. He graduated from the Faculty of Biology of the Shevchenko Kiev State University with a diploma in Biology–Zoology. He defended his PhD thesis “Fauna of Lepidoptera of the Middle Dnieper Valley” (1963) and his doctoral (habilitation) dissertation “Visual analysis of space in insects” (1981), both in entomology. For over 40 years, he has been working at the Schmalhausen Institute of Zoology of the Ukrainian National Academy of Sciences, where he currently holds the position of leading researcher.

Professor Fransevich made a number of important discoveries in the studies of insects. In particular, he discovered the ability of animals to recognize random two-dimensional images by their texture. He showed astro-orientation in Coleoptera during homing and identified structural elements of the olfactory center in the insect brain (glomeruli of the deutocerebrum) by morphological characteristics. He discovered, described and experimentally studied a type of proprioceptor (arcular organ) in Coleoptera. He demonstrated the spatial stability of visual orientation behind local and astro-landmarks in insects during homing on inclined surfaces. He proposed an orientation model using polarized sky light based on a standard polarization sensitivity direction map embedded in the retinal structure and demonstrated the spatial stability of topological signs of visual key stimuli in insects.

Leonid Frantsevich was the first to use the skeletal model of the kinematic system of walking insects for the purpose of describing and analyzing movements, solving the inverse kinematics problem for reconstructing the joint angles, which are not directly observed. Using inverse kinematics methods, he studied the kinematics of locomotor maneuvers in walking insects: turns on a plane, overturns, walking on thin rods, turning at the end of a thin rod, as well as the kinematics of opening–closing elytra in Coleoptera. He contributed to the research on the kinematics and mechanism of deployment of the arolium (a sticky pad at the insect pretarsus) and the role of pre-stressed structures in this process. Having studied the mechanics of the composed middle coxa in dipterans, Leonid Frantsevich showed that this structure is a marker of the body segment to which the leg is attached and discovered manifestations of homeosis (the appearance of a structure in another body segment) in certain dipteran taxa.

From autumn 1986 to 1988, Leonid Frantsevich headed the work of Kiev zoologists in the Chornobyl NPP site and the Exclusion Zone. At that time, his research developed in two directions: radioecology and general ecology. He calculated the volume of the removal of the radionuclides from the Exclusion Zone by migratory birds and then proposed an integral estimate of the offset as a product of three quantities. The resulting estimate turned out to be insignificant in comparison with the total removal of radionuclides outside the zone and did not require specific countermeasures.

In 1989–1994, Leonid Frantsevich and his colleagues carried out a wide bioindication of 90Sr pollution of water bodies and land on the basis of the beta radioactivity of mollusk shells. Maps of 90Sr pollution of the Kiev region and rivers of the Dnieper basin were compiled. The experience of data generalization for multi-species collections was used to reconstruct the radioactive pollution of various species of wild animals based on the study of a few representative species, which are accepted as comparison standards. This standardization made it possible to depict the radionuclide contamination of wild animals on a map (2000). Based on the methods of processing multicomponent collections, Leonid Frantsevich created the first model for optimizing the permissible levels of radionuclides in food (1997).

Leonid Frantsevich was the first to draw attention to the fact that, in most of the exclusion and resettlement zones (over 98% of the total area—about 3000 km^2^), the course of events in biocenoses was determined not by the harmful effect of radiation, but rather by the removal of anthropogenic pressure on wildlife after the evacuation of the population, eliminating large-scale engineering interventions. Research and accounting of general ecological patterns were needed for the management of the alienated territories. He proposed the concept of a mosaic reserve of the Exclusion Zone with the allocation of scientifically or nature-protected lands. The principle of the mosaic reserve was approved by the Scientific and Technical Council under the Administration of the Exclusion Zone.

Leonid Frantsevich has been very successful and productive (over 150 original publications and related books, the most significant of which are “Visual analysis of space in insects” (1980), “Spatial orientation of animals” (1986) and “Animals in the radioactive zone” (1991). During his career, he has received several awards for his many contributions to science, including the State Prize of the USSR (1987), the State Prize of the Ukraine (2004) and being elected as a corresponding member of the Ukrainian National Academy of Sciences (1990), to name a few.

We organized this Special Issue in honor of Professor Frantsevich’s distinguished scientific career over the past 60 years. This Special Issue consists of original research articles and review articles related to the functional morphology and biomechanics of insects, his favorite topic.
